# Modification of Gas6 Protein in the Brain by a Functional Endogenous Tissue Vitamin K Cycle

**DOI:** 10.3390/cells13100873

**Published:** 2024-05-18

**Authors:** Nadide Aydin, Bouchra Ouliass, Guylaine Ferland, Sassan Hafizi

**Affiliations:** 1 School of Medicine, Pharmacy and Biomedical Sciences, University of Portsmouth, Portsmouth PO1 2DT, UK; 2 Département de Nutrition, Université de Montréal, Montréal, QC H3T 1J4, Canada

**Keywords:** Gas6, TAM receptor, vitamin K, warfarin, glia, microglia, γ-carboxylation, γ-carboxyglutamic acid, neuroinflammation, neurodegenerative disease, nutrition

## Abstract

The TAM receptor ligand Gas6 is known for regulating inflammatory and immune pathways in various organs including the brain. Gas6 becomes fully functional through the post-translational modification of multiple glutamic acid residues into γ-carboxyglutamic in a vitamin K-dependent manner. However, the significance of this mechanism in the brain is not known. We report here the endogenous expression of multiple components of the vitamin K cycle within the mouse brain at various ages as well as in distinct brain glial cells. The brain expression of all genes was increased in the postnatal ages, mirroring their profiles in the liver. In microglia, the proinflammatory agent lipopolysaccharide caused the downregulation of all key vitamin K cycle genes. A secreted Gas6 protein was detected in the medium of both mouse cerebellar slices and brain glial cell cultures. Furthermore, the endogenous Gas6 γ-carboxylation level was abolished through incubation with the vitamin K antagonist warfarin and could be restored through co-incubation with vitamin K1. Finally, the γ-carboxylation level of the Gas6 protein within the brains of warfarin-treated rats was found to be significantly reduced ex vivo compared to the control brains. In conclusion, we demonstrated for the first time the existence of a functional vitamin K cycle within rodent brains, which regulates the functional modification of endogenous brain Gas6. These results indicate that vitamin K is an important nutrient for the brain. Furthermore, the measurement of vitamin K-dependent Gas6 functionality could be an indicator of homeostatic or disease mechanisms in the brain, such as in neurological disorders where Gas6/TAM signalling is impaired.

## 1. Introduction

Growth arrest-specific protein 6 (Gas6) and protein S (Pros1) are homologous γ-carboxyglutamic acid (Gla) domain-containing proteins that are ligands for the TAM (Tyro3, Axl, Mer) subfamily of receptor tyrosine kinases (RTKs). Upon ligand engagement, TAM receptors activate signalling pathways for different cellular processes such as cell survival, proliferation, migration and phagocytosis [1,2] the stimulation of cell growth, the inhibition of apoptosis [3,4], the stimulation of platelet-involved haemostasis [5] and the modulation of inflammation [6]. Increasing evidence points to a significant role for Gas6 in the nervous system. Gas6 was shown to protect oligodendrocytes from TNF-α toxicity [7] and cortical neurons from amyloid-induced death [8]. In a cuprizone-induced demyelination model, Gas6-deficient mice displayed lower oligodendrocyte survival, increased cell loss, and a reduction in overall myelination [9,10]. Therefore, these data suggest that Gas6/TAM signalling might be a promising candidate for new therapeutic treatment options in multiple sclerosis (MS) or other neurodegenerative diseases.

Gas6 is a multimodular protein which undergoes a number of post-transcriptional modifications, the most significant being a process that utilises vitamin K. As a broad group, vitamin K-dependent proteins (VKDPs) are involved in blood coagulation, bone metabolism, vascular calcification, the regulation of cell proliferation and signal transduction [11]. Vitamin K, as hydroquinone (KH_2_), is a cofactor for the enzyme gamma-glutamyl carboxylase (GGCX), which oxidises it to vitamin K 2,3-epoxide (KO) simultaneously to the γ-carboxylation of proteins in the endoplasmic reticulum (ER) [12]. The enzyme VKORC1 then reduces KO to KH_2_ using the enzyme’s two active site cysteine residues; this reduction is sensitive to warfarin inhibition. VKORC1L1, the product of a *VKORC1* gene paralogue, has also been shown to perform this same function particularly in extrahepatic tissues [13,14,15]. The NAD(P)H:quinone oxidoreductase 1 (NQO1) has also been suggested to reduce vitamin K quinone; however, it is not thought to play a significant role in the vitamin K cycle in comparison to VKORC1 [16,17,18]. This post-translational process is involved in the biological activation of VKDPs, of which Gas6 is a member. The resulting Gla residues in VKDPs are able to form calcium-binding groups via chelation [19], which is essential for binding to membrane phospholipids and modulating the protein’s physiological function [20,21]. In the case of Gas6, this translates to its ability to activate TAM receptors. Therefore, it would be expected that vitamin K also plays an important role in Gas6/TAM signalling and function, which moreover would be sensitive to inhibition by warfarin [1,2,22].

The liver and peripheral organs have different carboxylation processes. In particular, menaquinones (MKs) appear more important for peripheral carboxylation than phylloquinone (PK), which the liver appears to primarily utilise [23,24]. The human body is not able to store a high amount of vitamin K, which is rapidly depleted. Therefore, it is recycled through the vitamin K cycle [25], through which it can be reused many times for the purposes of protein carboxylation by GGCX. All forms of vitamin K have in common a core ring structure of 2-methyl-1,4-naphthoquinone (menadione; K3). Phylloquinone (2-methyl-3-phytyl-1,4-naphthoquinone) is mainly synthesised in green vegetables, whereas menaquinones (2-methyl-3-(all-trans-polyprenyl)-1,4-naphthoquinone) are mostly of bacterial origin [26]. However, menaquinone 4 (MK-4) is also able to be produced from dietary PK and stored largely in extrahepatic tissues, including notably in the brain [27]. This conversion is carried out by the enzyme Ubi prenyltransferase domain-containing protein 1 (UBIAD1), which has both a modest side-chain cleavage activity for phylloquinone and a significant prenylation activity for menadione [28].

In human studies, vitamin K status has been associated with cognitive functions. When investigated in cognitively intact elderly individuals, higher serum vitamin K (phylloquinone) concentrations were correlated with enhanced verbal episodic memory and recollection tests [29]. In a group of geriatric patients, those in the highest tertile of dietary phylloquinone intake reported better global cognition and behavioural rating [30]. In a recent report on post-mortem brains, higher levels of MK-4 were associated with lower odds of mild cognitive impairment and dementia. Further investigation of neuropathologically defined outcomes showed that increased brain MK-4 concentrations were associated with lower levels of neurofibrillary tangle density, high Braak stage, and the presence of Lewy bodies [31]. In addition, vitamin K has been shown to have anti-inflammatory properties: MK-4 was shown to inhibit the upregulation of inflammatory cytokines as well as NF-κB signalling during the microglial inflammatory response [32].

Given the independent lines of information indicating beneficial roles for both Gas6 and vitamin K during neuroinflammation, it is of interest to determine a direct link between these two molecules specifically within the brain. Here, we report for the first time the presence of a functional vitamin K machinery in the rodent CNS in vitro and in vivo, which can be regulated by exogenous vitamin K and warfarin in opposing ways to influence the γ-carboxylation of endogenous Gas6. 

## 2. Materials and Methods

### 2.1. Primary Mouse Glial Cell Cultures

All experiments involving animals were conducted under the stipulations of the UK Animals Scientific Procedures Act 1986 and approved by the award of a UK Home Office Project Licence (licence number PC2238199) as well as by the institutional ethics committee. Animals were killed under Schedule 1 by cervical dislocation. C57/BL6 mice (Charles River, Margate, UK) at different ages (embryonic day (E) 14.5, postnatal day (P) 0, P7, P14 and adult) were used. 

Primary glial cells were derived according to a previously published protocol [33]. Briefly, neocortex from neonatal mice (P0–2) were dissected and dissociated by mechanical trituration through a horse serum-coated (HS, Gibco, Fisher Scientific, Loughborough, UK) glass capillary pipette in cold media containing 10% foetal bovine serum (FBS, Lonza, Slough, UK), 10% HS and 1% penicillin/streptomycin (P/S; Fisher Scientific), named ‘20% medium’. The cell suspension was centrifuged at 20,000× *g* for 10 min at room temperature (RT). The cells were resuspended in 20% medium and seeded into sterile T75 flasks pre-coated with poly-D-lysine (0.01 mg/mL, Fisher Scientific) (mixed cultures were seeded onto pre-coated 24-well plates) and incubated at 37 °C with 5% CO_2_ for 10–14 days, after which they were ready for the successive removal of microglia and astrocytes. Microglia were isolated by subjecting the flasks to orbital shaking at 300 rpm for 3 h at 37 °C and removal of the medium containing the detached cells. The flasks were then replenished with fresh medium and shaken for a further 21 h. The media with further detached cells were removed, leaving pure astrocytes attached as a monolayer to the flasks. As previously described [34], immunofluorescence staining for glial cell markers Iba1 (microglia) and GFAP (astrocytes) verified the identities of the different cultures and their purities at ≥95%.

Cells were plated onto plastic plates coated with poly-D-lysine, at cellular densities of approximately 160,000 cells/cm^2^ for microglia and 80,000 cells/cm^2^ for astrocytes, and incubated at 37 °C with 5% CO_2_. Microglial cells were cultivated in 20% serum medium, while astrocytes were maintained in medium containing 10% serum only. The cells were allowed to adhere for a period of 24 h prior to their utilisation in experiments. In inflammatory agent stimulation experiments, cells were first cultured for 24 h in low-serum medium before incubation for 4 h and 8 h with various test agents as follows: lipopolysaccharide (LPS; 10 ng/mL), IL-4 (10 ng/mL), vitamin K1 (100 μM), vitamin D3 (100 nM) and dexamethasone (100 nM) (Sigma, Gillingham, UK). In another set of experiments, cells were pre-incubated with warfarin (3.24 μM) or vitamin K1 (11 μM) for 24 h, followed by medium replacement with 2% serum medium containing either warfarin or vitamin K1 for a further 48 h. After the 72 h period was over, the cell-conditioned medium was collected for the analysis of Gas6 by specific ELISA assays (below).

### 2.2. Mouse Organotypic Cerebellar Slice Culture

Organotypic cerebellum brain slice cultures were prepared according to previously published protocols [35,36,37,38]. In brief, cerebella were derived from P10–P12 mice and separated from the rest of the brain. The cerebellum was dissected in ice-cold minimum essential media (MEM) supplemented with Glutamax (Gibco) until taken for cutting sagittal slices of 300 µm thickness using a McIlwain Tissue chopper. The separated slices were transferred onto humidified sterile culture membrane inserts with pore sizes of 0.4 µm (Merck Millipore, Watford, UK). Tissue slices on membrane inserts were cultured on a liquid layer of medium containing 50% MEM media (Gibco), 25% HS, 25% Earle’s Balanced Salt Solution (EBSS) (Gibco) supplemented with 130 mM D-glucose (Sigma) and 1% P/S. The cultures were first maintained at 37 °C in 5% CO_2_ for 24 h prior to being used for in vitro experiments. After 24 h, the medium bathing the tissue was replaced with serum-free medium, into which treatments were added including vitamin K1 (100 µM) or warfarin (25 µM). After 72 h incubation, the culture medium was analysed for released Gas6 protein by ELISA assays for both total mouse Gas6 and Gla-Gas6 (below).

### 2.3. Protein Extraction from Mouse Tissues and Isolation of Mouse Liver and Brain Microsomes

Mouse tissues were dissected out, minced with fine scissors and placed in ice-cold RIPA buffer with added protease inhibitor cocktail (Fisher Scientific). Tissues were mechanically homogenised with a pestle motor, and the homogenates underwent multiple successive rounds of vortexing, with incubations on ice in between each round. The tubes were finally centrifuged at 24,100× *g* for 10 min at 4 °C, and the supernatant was transferred into clean collection tubes and the samples stored at −80 °C until required.

The isolation of mouse brain microsomes was performed according to a modification of the method of Ravindranath et al. [39]. Briefly, isolated brains from 3-month-old adult WT mice were homogenised in a homogenisation buffer (0.1 M Tris, 0.1 mM DTT, 1.15% potassium chloride, 20% (*v*/*v*) glycerol, protease inhibitor cocktail, pH 7.4). The homogenates underwent successive rounds of centrifugation at 17,000× *g* for 30 min at 4 °C. The supernatants separated from pellets in these initial centrifugation steps were pooled and finally underwent ultracentrifugation at 100,000× *g* (CP100NX, Hitachi, Eppendorf Himac Technologies Co., Ltd., Hitachinaka, Japan) for 1 h at 4 °C. The pellet was suspended in a minimum quantity of the homogenisation buffer and aliquots were stored at −80 °C until required. The isolation of mouse liver microsomes was performed according to the method of Samanta et al. [40] with modifications. Pure guinea pig liver microsome preparations were provided in-house by the department, where they were routinely used for drug metabolism assays. Protein concentration in samples was determined by the bicinchoninic acid protein assay using BSA as standard.

### 2.4. RT-qPCR

The extraction of total RNA from animal tissues was performed using the Monarch^®^ Total RNA Miniprep Kit (New England Biolabs; Hitchin, UK) according to the manufacturer’s instructions. This included an additional pre-step for tissue homogenisation. Rodent tissue at up to 30 mg wet weight were mechanically disrupted and homogenised using a pestle motor in round-bottomed collection tubes containing 1x RNA protection reagent. Total RNA from primary glial cell cultures was extracted using the RNeasy^®^ Mini Kit (Qiagen, #74106, Hilden, Germany) according to the manufacturer’s protocol. Briefly, depending on the number of cells, a corresponding volume of RNA lysis buffer was added to the cells, which were mechanically disrupted within the plates and then removed. In the final step of all extraction protocols, RNA was eluted in nuclease-free water by centrifugation. RNA purity and concentration were measured using a spectrophotometer (ND-1000; NanoDrop Technologies, Wilmington, DE, USA).

Total RNA was reverse-transcribed into complementary DNA (cDNA) using the High-Capacity cDNA Reverse Transcription Kit (Applied Biosystems^TM^, Forster City, CA, USA). All cDNA samples were used for mRNA expression analysis by real-time PCR, using gene-specific primers and TaqMan™-style fluorescent hydrolysis probes added to a ‘master’ reaction mix (FastStart Essential DNA Probes Master; Roche, Burgess Hill, UK). Pre-designed primer/probe mixes (Integrated DNA Technologies (IDT); Leuven, Belgium) were used for all genes analysed by qPCR. Each sample was also subjected to qPCR reaction for a reference or ‘housekeeping’ gene, which was *Gapdh*. All data were analysed using the 2^−ΔCt^ method for calculating the relative gene expression level or the 2^−ΔΔCt^ method for fold change in gene expression as previously reported [41].

### 2.5. SDS-PAGE and Western Blotting

For protein extraction, cultured cells, microsome preparations and tissues were lysed in ice-cold RIPA buffer (150 mM NaCl, 1% Triton X-100, 0.5% sodium deoxycholate, 0.1% SDS, 50 mM Tris pH 8.0) supplemented with protease inhibitors. Tissues were mechanically homogenised with a pestle motor. Lysates were applied to sodium dodecyl sulphate polyacrylamide gel electrophoresis (SDS-PAGE). The separated proteins were subsequently transferred onto an activated polyvinylidene fluoride membrane (Millipore, Nottingham, UK) using a wet transfer method. Membranes were incubated in blocking buffer (3% non-fat dry milk in Tris-buffered saline-Tween 0.1% (TBS-T) for 1 h at RT. Afterwards, the membranes were exposed to primary antibodies, appropriately diluted in the same blocking buffer, and left to incubate overnight at 4 °C. The primary antibodies (and dilutions) used were as follows: GGCX (rabbit polyclonal, 1:2000, Proteintech, Manchester, UK), VKORC1 (rabbit polyclonal, 1:1000, Novus Biologicals, Centennial, CO, USA), β-actin (mouse monoclonal, 1:1000, Cell Signalling Technology, London, UK). Afterwards, the membranes were washed three times for 5 min with TBS-T, followed by incubation with corresponding horseradish peroxidase (HRP)-conjugated secondary antibodies for 2 h at RT. The secondary antibodies (and dilutions) used were anti-rabbit IgG (H + L) HRP (1:5000, Thermo Fisher Scientific, Loughborough, UK) and anti-mouse IgG (H + L) HRP (1:2000, Seracare Life Sciences Inc., Milford, MA, USA). Following a subsequent round of washing, the membranes were incubated with an enhanced chemiluminescence (ECL) development reagent (Luminata Forte, Millipore) and images captured with a chemiluminescence CCD camera (ImageQuant LAS500, GE Healthcare Life Sciences, Little Chalfont, UK). The software ImageJ v1.8 [42] was used for the densitometric quantification of Western blot band intensities.

### 2.6. Immunoassays for Total and Carboxylated Gas6

Gas6 proteins, as well as Gla residues in Gas6, were quantified in samples using specific sandwich ELISAs. Total Gas6 ELISA was performed according to the manufacturer’s instructions (R&D Systems, Bio-Techne, Minneapolis, MN, USA). The Gla-Gas6 ELISA was developed in-house for the detection of specifically γ-carboxylated (Gla) residues in Gas6 in samples. The ELISA was confirmed for specificity against the Gas6 protein where the γ-carboxylation was modified through opposing treatments (vitamin K, warfarin), and the anti-Gla antibody was verified for specificity by Western blotting of Gas6 under the same opposite conditions (Supplementary Figure S3). Briefly, a 96-well microplate with a high protein binding capacity (Maxisorp Nunc-Immuno plate, Thermo Fisher Scientific was coated with an anti-mGas6 antibody (0.2 µg/mL, R&D Systems; 1 µg/mL for Gla-Gas6 assay) diluted in a coating buffer (30 mM Na_2_CO_3_, 200 mM NaHCO_3_, pH 9.0), and the plates were incubated overnight at 4 °C. The plates were washed three times with 400 µL per well of washing buffer. The plate was then blocked with 1% BSA (2% BSA for Gla-Gas6 ELISA) for 2 h at RT. After the washing step, appropriately diluted samples in PBS (PBS, 0.1% BSA for Gla-Gas6 ELISA) were added and incubated overnight at 4 °C. The samples were removed and the plate was again washed, after which the detection antibody, biotinylated anti-mGas6 (0.2 µg/mL, R&D Systems), was added and incubated for 2 h at RT. After washing, streptavidin-HRP (DY998, R&D Systems) was added and incubated in the dark for 20 min. For the Gla-Gas6 ELISA, the detection antibody was mouse anti-Gla (mAb 3570; Biomedica Diagnostics, Stamford, CT, USA) which was diluted (0.25 μg/mL) in dilution antibody buffer (PBS, 0.1%, BSA%, 0.05% Tween-20) and incubated for a minimum of 3 h at RT. The solution was removed and anti-mouse HRP in dilution antibody buffer (1:2000) was added to each well and incubated for a minimum of 1.5 h at RT.

The final step was to wash the plate and to add a substrate solution, which was a 1:1 mixture of Colour Reagent A (H_2_O_2_) and Colour Reagent B (3,3′,5,5′-tetramethylbenzidine; TMB) (Pierce^TM^ TMB Substrate Kit, Thermo Fisher Scientific), and the plate was protected from direct light during colour development. A stop solution (2M H_2_SO_4_,) was added to the wells, and optical density (OD) was measured with a microplate reader (SpectraMax i3x, Molecular Devices, San Jose, CA, USA) at wavelengths 450 nm and 570 nm. In all ELISA assays, the measured values were blanked to eliminate any interference from background signals. To perform blanking for each treatment, the OD 570 nm value was subtracted from the 450 nm value, and from this was subtracted the OD value of the zero standard, i.e., buffer or medium only, as appropriate.

### 2.7. Statistical Analysis

All experiments were conducted a minimum of three times, unless otherwise stated. All data are shown as the mean ± standard error of the mean (SEM) with statistical significance based on the following criteria: * *p* < 0.05, ** *p* < 0.01, *** *p* < 0.001 and **** *p* < 0.0001, unless otherwise stated in the accompanying figure legends. Analysis of variance (ANOVA) (parametric) with a post hoc Tukey test was used as a statistical test for multiple comparisons within groups or with one control group. Statistical significance was determined for unpaired comparisons using Welch’s *t*-test (parametric) or unpaired *t*-test (parametric). For a comparison of independent unpaired groups (non-parametric), the Mann–Whitney test was used. Statistical analysis and graphs were designed using Prism software v.9.0.2 (GraphPad Software Inc., San Diego, CA, USA).

## 3. Results

### 3.1. Gene Expression of TAM Receptors and TAM Ligands in Mouse Tissues and Brain Glial Cells

**Figure 1:**
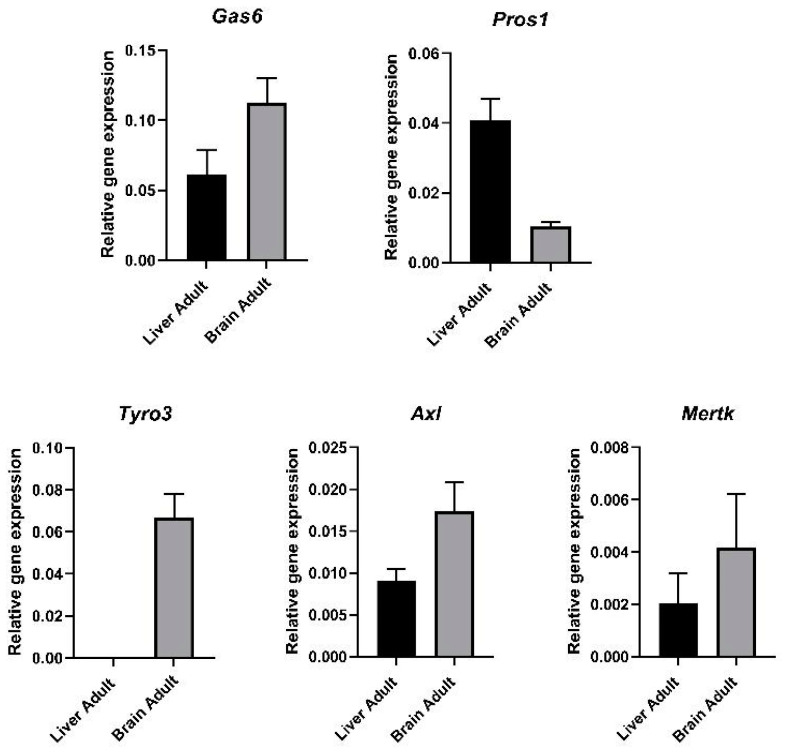
Gene expression of TAM ligands and TAM receptors in adult mouse tissues. RT-qPCR analysis of relative gene expression in adult mouse liver and brain of TAM ligands Gas6 and Pros1 and TAM receptors Tyro3, Axl and Mertk. Relative gene expression was analysed by using 2^−ΔCt^ method, using *Gapdh* as housekeeping gene (mean ± SEM, *n* = 3 tissues).

RT-qPCR was used to determine the mRNA expression of the three TAM RTKs and the two TAM ligands in the adult mouse brain as well as liver for comparison. The results showed that Gas6 and Pros1 are expressed in both organs of the adult mouse although with notable differences (Figure 1). Gas6 mRNA expression was roughly two-fold higher in the brain than in the liver, whilst in contrast, Pros1 expression was roughly four-fold higher in the liver than in the brain. There were also organ-specific differences in expression among the TAM receptors. All three TAMs were expressed in the mouse brain, whereas in the liver, only Axl and Mertk were expressed and at slightly lower levels than in the brain. Furthermore, an examination of raw amplification data from the qPCR runs indicated that Tyro3 was notably highest in expression in the brain amongst the TAMs.

**Figure 2:**
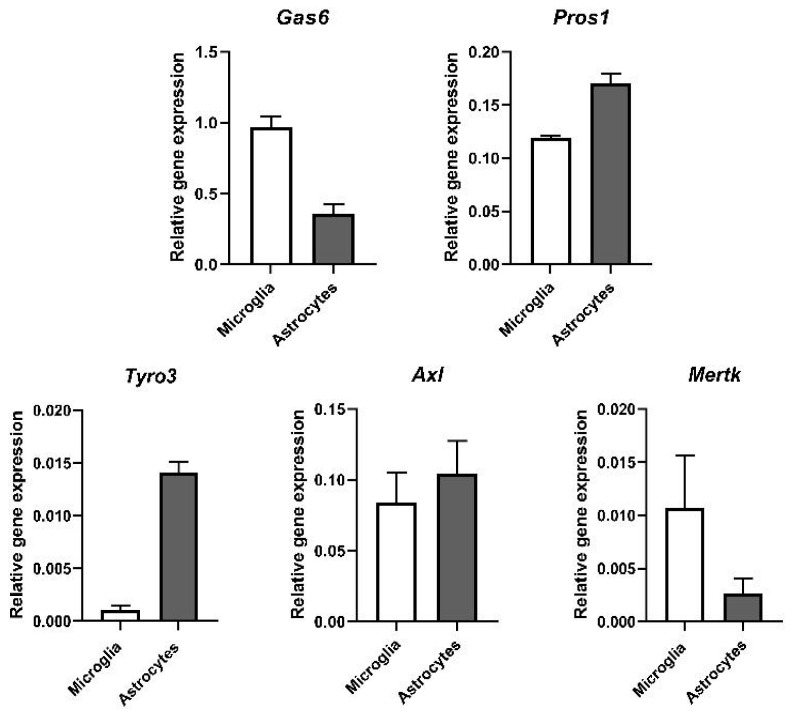
Gene expression of TAM ligands and TAM receptors in mouse brain glial cells. RT-qPCR analysis of gene expression of TAM ligands Gas6 and Pros1 and TAM receptors Tyro3, Axl and Mertk in primary cultures of astrocytes and microglia. Relative gene expression was analysed by using 2^−ΔCt^ methods, using *Gapdh* as housekeeping gene (mean ± SEM; *n* = 3 cultures).

Having determined the expression of the TAM ligands and receptors in the mouse brain, the mRNA expression of the same genes was also determined in pure primary mouse brain microglia and astrocytes in culture. Both TAM ligands were found to be expressed in both glial cell types; Gas6 expression was two-fold higher in microglia than in astrocytes, whereas Pros1 expression was slightly higher in astrocytes (Figure 2). As regards the TAM receptors, Tyro3 appeared to be virtually restricted to astrocytes amongst the two cell types, whereas Mertk was more prominent in microglia than in astrocytes. Axl expression was comparable across both cell types.

### 3.2. Expression of Vitamin K Cycle Enzyme Genes in the Mouse Brain during Embryonic and Postnatal Development and in Brain Glial Cells

**Figure 3:**
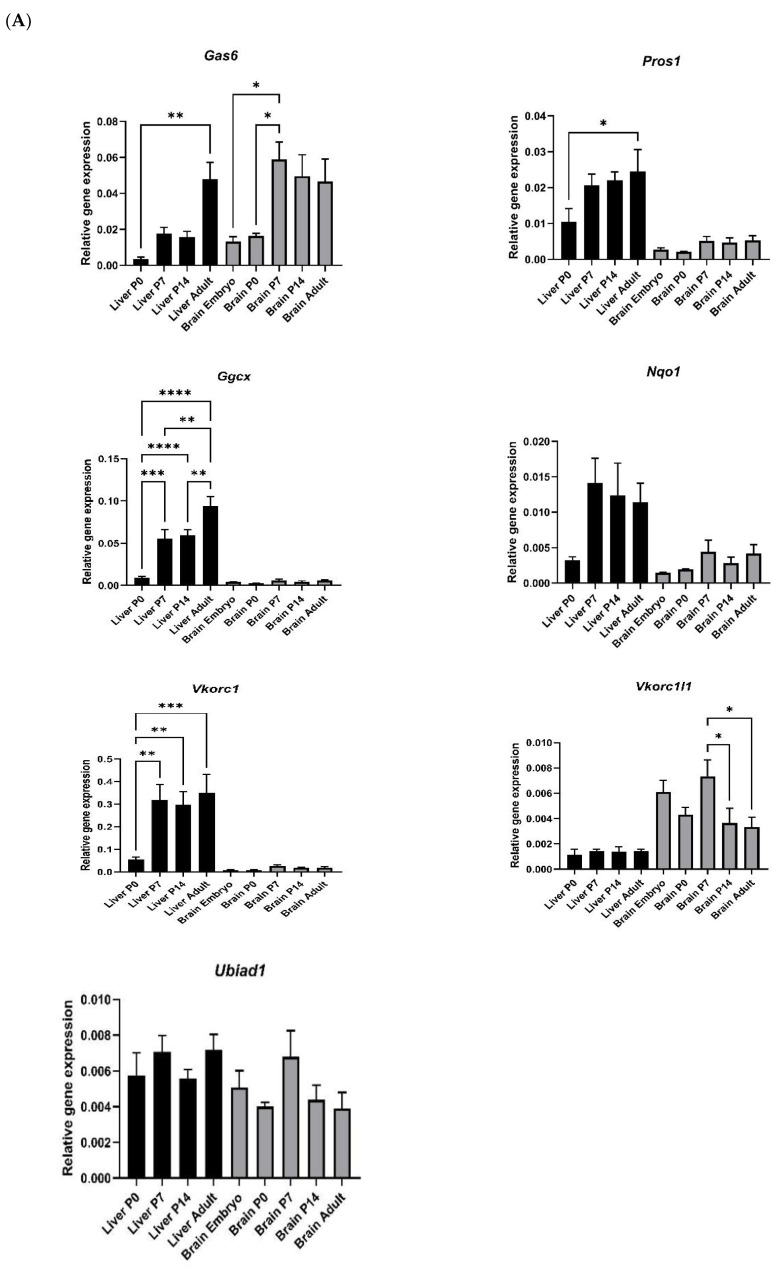

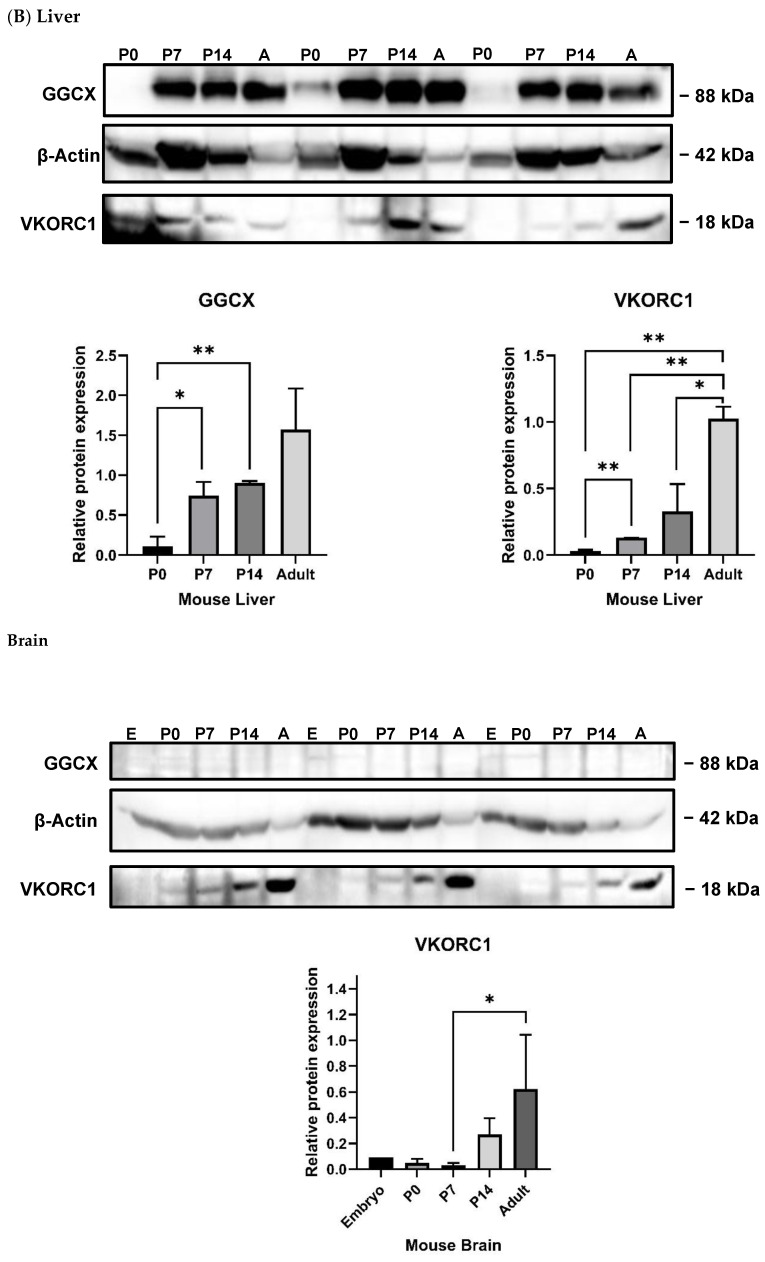
Analysis of gene and protein expression of vitamin K cycle enzymes in mouse liver and brain during postnatal development. (**A**) Relative gene expression was determined by RT-qPCR and data was analysed by 2^−ΔCt^ method, using *Gapdh* as housekeeping gene (mean ± SEM; *n* = 4 tissues; brain embryo (E) *n* = 3). ANOVA with Tukey’s multiple expression multiple comparison post hoc analysis * *p* < 0.05, ** *p* < 0.01, *** *p* < 0.001, **** *p* < 0.0001. (**B**) Western blot detection of GGCX and VKORC1 proteins in extracts from liver (P0—adult (A); upper blot) and brain (E–A; lower blot). Accompanying graphs show protein quantification by band densitometry analysis. Data are mean ± SEM protein expression normalised against β-Actin as loading control protein. Data underwent unpaired *t*-test; * *p* < 0.05, ** *p* < 0.01 between samples as indicated (*n* = 3).

As many VKDPs and vitamin K cycle components in the liver are predominantly upregulated in their expression after birth, it was of interest to investigate whether the same occurs in the brain. Therefore, we analysed the mRNA expression of vitamin K cycle enzymes and the TAM ligands in both mouse brain and liver tissues at different stages of development. In the liver, the mRNA for *Gas6*, *Pros1*, *Vkorc1* and *Ggcx* genes were all detected at significantly higher levels in postnatal tissues compared to P0 (Figure 3A). *Nqo1* was also increased although not significantly. In the brain, only *Gas6* showed significantly a higher expression in postnatal ages compared to P0. Although the qPCR data for both tissues are on the same graphs and axis scales for ease of display, the profiles for other genes in the brain, although less clear, also showed trends towards age-dependent increases. It should, however, be borne in mind that these two tissue profiles cannot be directly comparable as the housekeeping gene would not be expected to be expressed at similar levels in the different tissues. Also noteworthy was that *Vkorc1l1*, a paralogue of the *Vkorc1* gene, was the only gene that was consistently higher in the brain vs. the liver at all ages, whereas the opposite was true for all the other genes analysed. Another key difference was that Gas6 appeared to reach maximal expression levels in the liver only in adulthood, whereas in the brain, maximal expression had been reached by P7.

Protein levels across the ages, as analysed by Western blotting, showed some similarities but also differences to the gene expression profiles. In the liver, both GGCX and VKORC1 proteins steadily increased in their expression levels after age P0, reaching maximum expression that was significantly highest in adulthood (Figure 3B). In the brain, the VKORC1 protein also steadily increased in expression after age P0, reaching maximum levels in adulthood. However, GGCX was not detected, suggesting protein levels in crude brain extracts were below the sensitivity of the antibody (whereas it was detected in enriched brain microsomes (Supplementary Figure S1)).

**Figure 4:**
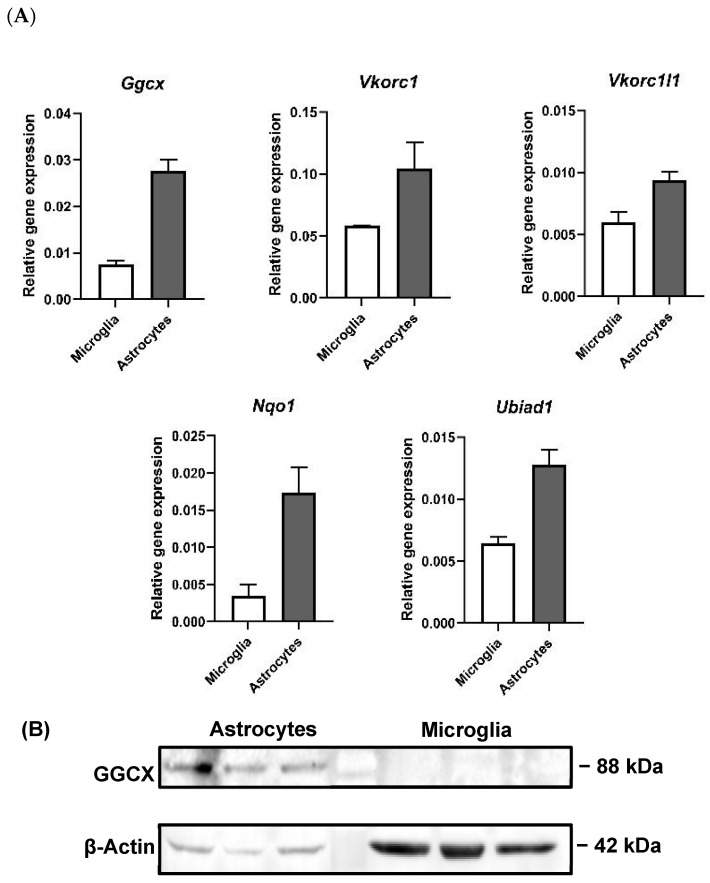
Gene and protein expression of vitamin K cycle enzymes in primary cultured mouse brain astrocytes and microglia. (**A**) Relative gene expression was analysed by using 2^−ΔCt^ method, using *Gapdh* as housekeeping gene (mean ± SEM; *n* = 3 cultures). (**B**) Western blot showing GGCX protein expression in astrocytes. β-Actin was used as protein loading control. *n* = 3 separate culture extracts loaded.

The qPCR data on mRNA expression are relative values, which may not necessarily directly correspond to protein expression; thus, both sets of data have greater value together. It was furthermore determined that all five vitamin K cycle enzyme genes were expressed in both cultured brain astrocytes and microglia (Figure 4A). Astrocytes showed higher expression levels for all genes. Only astrocyte cell extracts showed a detectable expression of GGCX protein at the same apparent molecular weight of 88 kDa as in whole tissues (Figure 4B). In addition, we analysed protein expression in microsomes isolated from both mouse livers and brains. These analyses showed, for the first time, the expression of the GGCX protein in enriched microsomes of the mouse brain (Supplementary Figure S1).

### 3.3. Regulation of Expression of Vitamin K Cycle Enzyme and TAM Ligand Genes by Exogenous Agents in Cultured Mouse Brain Glial Cells

**Figure 5:**
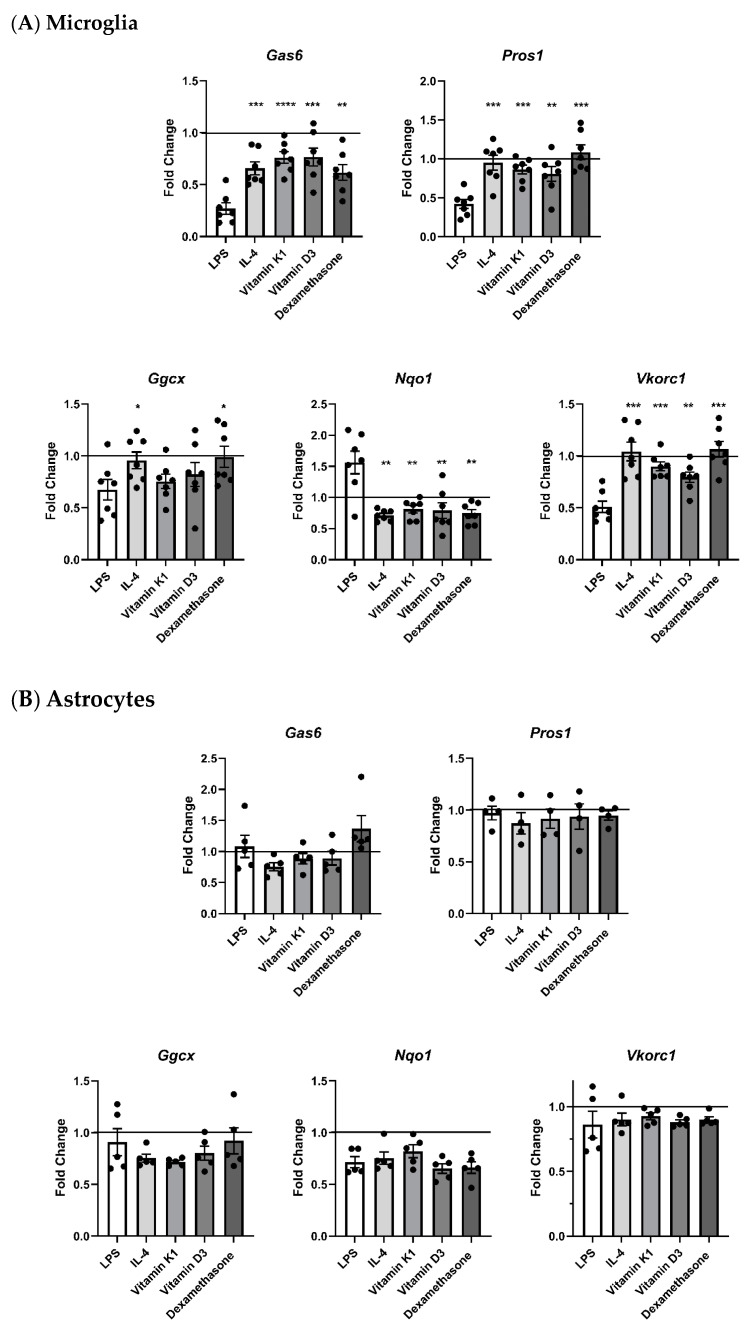
RT-qPCR analysis of mRNA expression of TAM ligands and vitamin K cycle enzymes in glial cells undergoing treatment with different agents. Expression of K cycle enzyme genes (*Ggcx*, *Nqo1*, *Vkorc1*) and *Gas6* and *Pros1* in pure primary cultures of (**A**) microglia and (**B**) astrocytes after 8 h incubation with agents. Relative gene expression was analysed by 2^−ΔΔCt^ method, using *Gapdh* as housekeeping gene (mean ± SEM, microglia *n* = 7 cultures, astrocytes *n* = 5 cultures (*n* = 4 for *Pros1*)). Horizontal line in each graph shows baseline expression in untreated cells, with fold changes displayed relative to that. Statistical significance was determined using Welch’s *t*-test with * *p* < 0.05, ** *p* < 0.01, *** *p* < 0.001, **** *p* < 0.0001 vs. LPS.

We then investigated the regulation of the expression of the TAM ligand and vitamin K cycle genes in brain glial cells in response to a variety of exogenous agents relevant to neuroinflammation. Mouse primary glial cells were incubated with various test agents as follows: LPS (10 ng/mL, Sigma), IL-4 (10 ng/mL), vitamin K1 (100 μM), vitamin D3 (100 nM) and dexamethasone (100 nM). LPS was selected to stimulate inflammatory signalling in the glial cells, whereas IL-4 functioned mainly as an anti-inflammatory cytokine. Vitamin K1 was used to explore a potential feedback mechanism for the K cycle component genes. Vitamin D3 and dexamethasone are both cholesterol derivatives with direct gene regulatory properties. For example, dexamethasone has been shown to upregulate VKDPs such as Gas6 as well as stimulate vitamin K-dependent γ-carboxylase activity [43]. Microglia were incubated with agents for 8 h, whereas astrocytes were treated for 4 h (Supplementary Figure S2) and 8 h separately. In microglia, when comparing both baseline gene expression and the expression with other test agents, LPS consistently altered the expression patterns of all the genes of interest (Figure 5A). LPS caused a significant downregulation of the expression of *Gas6*, *Pros1*, *Ggcx* and *Vkorc1* genes, whereas *Nqo1* expression was significantly increased. The astrocyte response to incubation with the test agents after 8 h showed no overall significant changes (Figure 5B), with the exception that LPS after 4 h caused a significantly higher expression of *Ggcx* compared to all other treatments (Supplementary Figure S2).

### 3.4. Warfarin Blocks the Functionality of the Vitamin K Cycle in Microglia and Astrocytes

**Figure 6:**
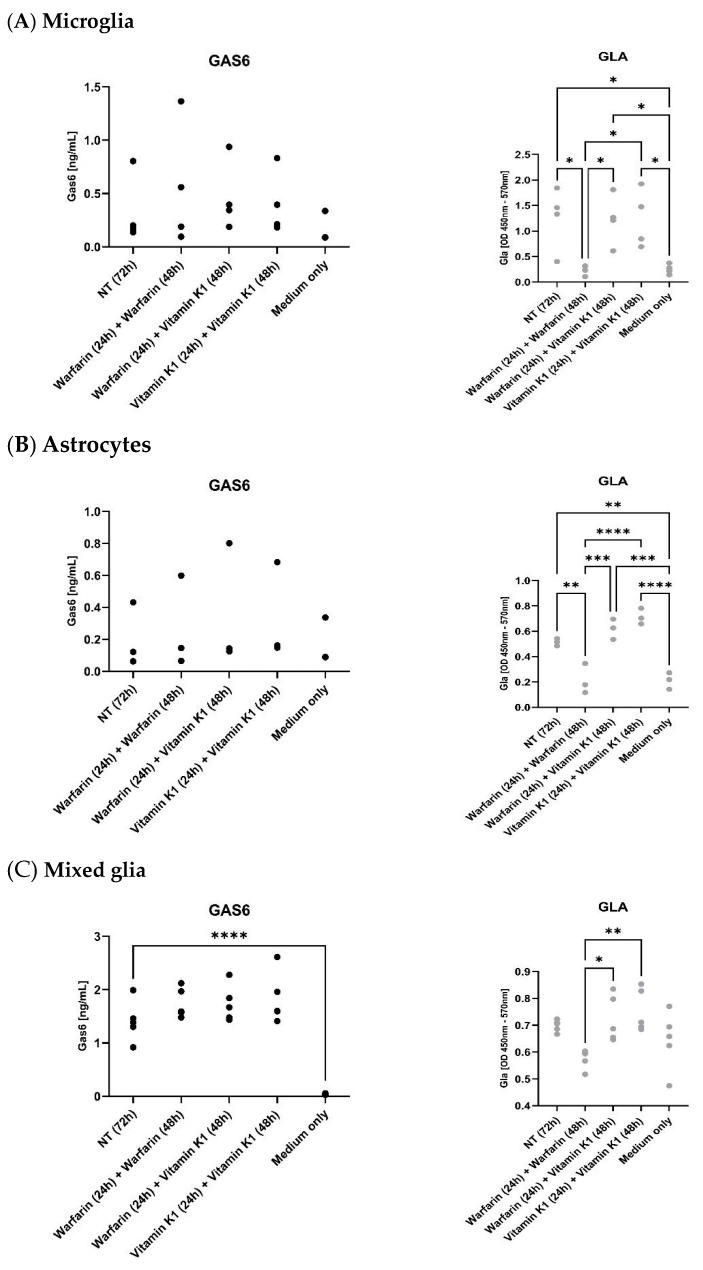
Mouse brain glial cells in culture release endogenous Gas6 protein that can be suppressed in its γ-carboxylation by warfarin and subsequently reversed by exogenous vitamin K1. Pure cultures of microglia (**A**), astrocytes (**B**) as well as mixed glia (**C**) were pre-treated with warfarin (3.24 μM) or vitamin K1 (11 μM) for 24 h, followed by replacement with 2% serum medium with added warfarin or vitamin K1 for a further 48 h. ‘NT’ is conditioned medium that has been incubated with cells but with nothing extra added, whereas ‘medium only’ is medium that has not been incubated with cells. Media were analysed by specific ELISA assays for total mouse Gas6 protein (left graph, black dots) and Gla-Gas6 (right graph, grey dots). Statistical significance was determined by one-way ANOVA with Tukey’s multiple comparisons test, * p < 0.05, ** p < 0.01, *** p < 0.001, **** *p* < 0.0001 for comparisons indicated by lines (microglia *n* = 4, astrocytes *n* = 3, mixed glia *n* = 5 on separate cultures).

We next investigated the effects of exogenous vitamin K1 and warfarin on the carboxylation status of the Gas6 protein secreted by glial cells. Experiments were conducted on separate cultures of pure microglia and astrocytes as well as mixed glia. The levels of both total Gas6 and exclusively γ-carboxylated Gas6 (Gla-Gas6) were measured separately using specific ELISA assays. The results show a clear competitive antagonism between vitamin K1 and warfarin in terms of the γ-carboxylation level of Gas6 released by the cells. In all three culture types, constant warfarin only exposure resulted in the complete abrogation of Gas6 γ-carboxylation (Figure 6). However, cells first pre-treated with warfarin followed by a replacement with vitamin K1 showed Gla-Gas6 levels equal to those of the cells constantly exposed to vitamin K1 alone. Therefore, added vitamin K1 completely antagonised the warfarin effect. All three glial culture types displayed the same pattern of results, showing that cellular Gas6 is regulated through a functional endogenous cellular vitamin K cycle. It was observed that the mixed glia cultures released markedly higher Gas6 levels than the pure cultures. This could be due to higher cell numbers as well as greatly enhanced Gas6 expression in the glial co-cultures, which may have had more favourable conditions and growth characteristics than the pure cultures. When comparing specific cell types, microglia released more Gas6 than astrocytes; this is apparent in the different scales of the *Y* axes across the three culture types.

### 3.5. Vitamin K Increases Gas6 γ-Carboxylation in Mouse Cerebellum

**Figure 7:**
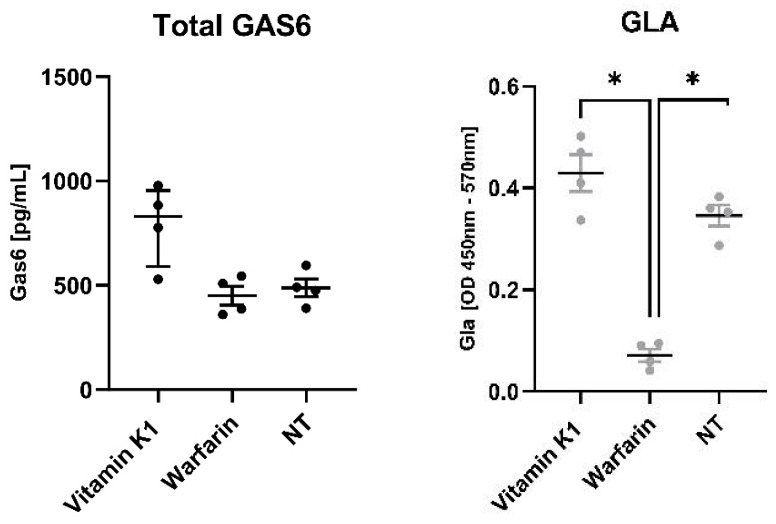
Effects of vitamin K1 and warfarin on levels of total and γ-carboxylated Gas6 released by mouse cerebellar slice cultures. γ-carboxylated Gas6 (right graph, grey dots) is significantly decreased by warfarin in comparison to vitamin K1 and control. Statistical significance was determined using Mann– Whitney test; * p < 0.05 for comparisons indicated by lines (*n* = 4 separate cultures).

The effects of vitamin K1 and warfarin were tested on the γ-carboxylation of natural endogenous Gas6 released from mouse cerebellar slice cultures. Warfarin profoundly reduced the Gla-Gas6 levels without affecting the total Gas6 levels (Figure 7). In contrast, vitamin K1 tended to slightly increase both total Gas6 and Gla-Gas6 levels above the control although not significantly. Therefore, through the observations on γ-carboxylated Gas6 release and the effect of warfarin, these results show that intact brain tissue possesses an active and functional vitamin K cycle and related GGCX activity.

### 3.6. Warfarin Suppresses γ-Carboxylation of Gas6 in Rat Brain Tissue Ex Vivo

**Figure 8:**
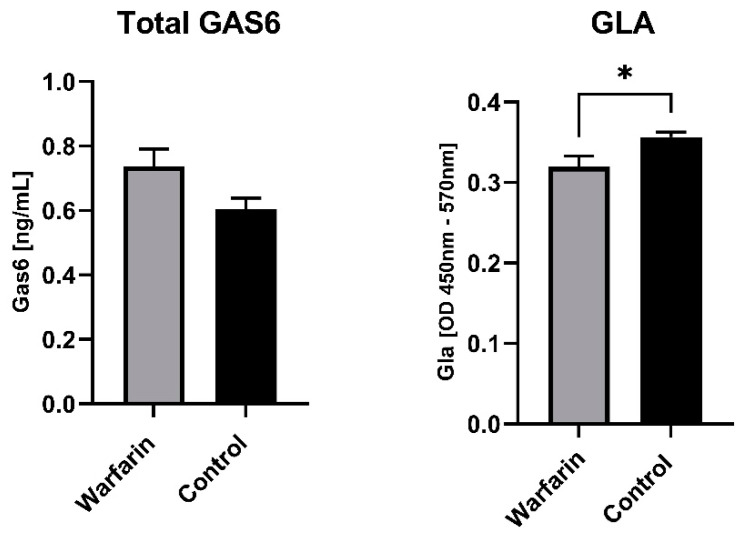
γ-carboxylated Gas6 protein is downregulated in the brains of warfarin-treated rats in vivo. Eight-week-old male Wistar rats were treated with 14 mg/kg per day with warfarin in the drinking water and subcutaneous vitamin K1 (85 mg/kg/day) injections three times per week for 10 weeks. Control animals were treated with normal water and injected with saline three times per week for 10 weeks in total. Control group were fed with AIN-93-based diet containing 750 μg vitamin K1/kg. The bar graphs show comparisons of control (black bars) vs. warfarin (grey bars) for levels of total Gas6 and Gla-Gas6. Statistical analysis was determined with unpaired *t*-test * p < 0.05 (*n* = 8 separate tissues).

Extracts from ex vivo brain samples, which had come from a study previously conducted by us [44], were analysed for Gas6 carboxylation. In the prior study, 8-week-old male Wistar rats had been treated with warfarin in their drinking water (14 mg/kg/day) together with subcutaneous vitamin K1 injections (85 mg/kg/day), three times per week for 10 successive weeks. Control animals were provided normal water and injected with saline over the same period. The diet for both groups was AIN-93-based containing 750 μg vitamin K1/kg diet. Equal amounts of the total protein from the tissue extracts were analysed by ELISA assays for the total Gas6 and Gla-Gas6 (Figure 8). There was no significant difference in the total brain Gas6 protein concentration between the control and warfarin groups. However, the γ-carboxylated Gas6 level was significantly lower in the warfarin group in comparison to the control. Therefore, this analysis showed that the in situ Gas6 within the brains of rats administered warfarin in vivo was less γ-carboxylated than that in the control animals.

## 4. Discussion

The many liver-derived coagulation factors that are vitamin K-dependent proteins (VKDPs) have been well studied for their functions, structures, molecular interactions and vitamin-K-dependent modification mechanisms. However, the expression and roles of VKDPs specifically in the CNS is an area that remains to be clarified. The focus of this study was on the expression and functional regulation of the VKDP and TAM ligand Gas6, as well as of multiple vitamin K cycle regulators, in the mouse brain.

Previously, we and others have shown that Gas6 is expressed in the brains of rodents and moreover that its expression increases in the early stages of brain development [45,46]. We confirmed this here as well as observing Gas6 expression in microglia and astrocytes. Pros1 is well known as a blood coagulation regulatory protein that is predominantly expressed in the liver [47]. Here, we observed a comparatively low expression of Pros1 in the mouse brain in comparison to its strong expression in the liver. We detected a strong gene expression of Mertk and Axl, but not Tyro3, in microglia, whilst in astrocytes, there was clear expression of both Tyro3 and Axl. These results concur with previous findings. In human and mouse microglia, Mertk was reported as highly expressed and Axl at lower levels, with Tyro3 being negligible [48,49]. By comparison, astrocytes showed similar expression levels of Tyro3 and Axl [49]. Our data also broadly concur with RNA-Seq data from an expression study of different cell types purified from a mouse cortex, highlighting in particular the stark contrast between astrocytes and microglia for Tyro3 expression, as well as the higher Gas6 expression in microglia (Supplementary Figure S4) [50,51]. Tyro3 has also been shown to be abundant in neurons [52]. Mertk and to a lesser extent Axl are required for the phagocytosis of apoptotic cells, as evidenced by prominent Mertk expression by all phagocytic macrophages [53,54,55]. Axl, moreover, is known as a negative feedback inhibitor of Toll-like and cytokine receptor signalling in dendritic cells and microglia [34,55]. 

Although the RT-qPCR does not provide for a direct comparison of expression levels across the different genes, we can gauge these to a degree by comparing the *Y* axes between them, which reflects the degree of amplifications in the reactions. Such analysis indicates that Tyro3 is substantially more highly expressed in the mouse brain than Mertk; whereas conversely, Tyro3 is absent in mouse liver whilst Mertk is expressed there.

Our findings on TAMs and TAM ligand expression in the CNS further inform our novel findings on the co-expression of the VKDP regulators. We observed the expression of vitamin K cycle enzyme genes in both microglia and astrocytes and additionally VKORC1 and GGCX at the protein level. Previous studies have utilised pure tissue microsome preparations for the study of ER-associated proteins, as has been conducted for GGCX and its enzymatic carboxylation activity in liver microsomes [56]. In the present study, we show for the first time the expression of GGCX proteins in adult mouse brain microsomes in addition to liver microsomes. The enrichment of microsomes for analysis, which entails the concentration of ER-associated proteins, of which GGCX is one, enabled the identification of the enzyme where it was below the immunoblot detection limit in whole brain extracts. Therefore, these observations together add to the evidence for a functional vitamin K cycle in ER-associated structures in the brain and consequently the production of VKDPs such as Gas6 that could be important for brain functions.

We were also interested in the expression of these components throughout early brain development as an indication of their functional importance for this organ. Gas6 gene expression increased in the brain and liver in line with early postnatal development, which is agreement with previous findings on both Gas6 and TAM receptors [45,57]. Here also, we obtained novel data on the expression of the vitamin K cycle enzyme genes in the mouse brain during development. Their expression profiles largely mirrored those in the liver, with expression peaking after birth, thus confirming their roles as not necessary for embryonic development but instead in homeostatic mechanisms in the postnatal brain [15,58]. In the liver, *Vkorc1* showed a strong increase in the first postnatal week that remained constant thereafter. The same trend was observed in the brain, although the levels were much lower relative to the liver. A noteworthy contrast was that *Vkorc1l1*, a paralogue of *Vkorc1*, was the only gene whose expression was higher in the brain than in the liver, with levels reaching a maximum during embryogenesis. These observations match previous reports of comparative *Vkorc1* and *Vkorc1l1* expression in a wide range of adult mouse tissues [13,14,15]. Therefore, together they suggest that in the brain, VKORC1L1 may play a more prominent role in the recycling of vitamin K from its oxidised to reduced form [58]. Furthermore, our data broadly match the expression profiles of the same genes in an RNA-Seq study of different mouse organs during development (Supplementary Figure S4) [50,59].

Our vitamin K cycle gene expression screen also included the *Nqo1* gene as it has in the past been suggested as an additional oxidoreductase potentially involved in vitamin K cycling. We observed a similar expression pattern for *Nqo1* as *Ggcx* and *Vkorc1* in the liver and the brain. However, an appreciable role for NQO1 in the vitamin K cycle is unlikely as more recent studies in cultured cells and knockout mice showed no detectable function for NQO1 in the classical vitamin K cycle and haemostasis [16,17,18]. Recently, ferroptosis suppressor protein1 (FSP1) was identified as the enzyme that reduces vitamin K to KH_2_ [60]. In contrast to the other genes, the *Ubiad1* gene showed no pattern of association with either organ or age, being expressed at similar levels in both organs at all ages. UBIAD1 has been shown to catalyse the conversion of phylloquinone to MK-4 in extrahepatic tissues [28]. UBIAD1 knockout mice die during embryogenesis [61], and our observations of maximal gene expression in embryonic tissues concur with this. UBIAD1 is found in the ER, but it is not inhibited by warfarin [62]. The findings that UBIAD1 may be a key factor involved in the metabolic production of MK-4 in the brain support the results of other studies that have shown MK-4 to be the predominant K vitamer in the rodent brain [63].

Having established the presence of the TAM ligands and vitamin K cycle components in the mouse brain and glial cells, we also enquired about the regulation of these genes. Lipopolysaccharide (LPS) is well established as a pro-inflammatory stimulant for microglia, acting through the upregulation of major pro-inflammatory cytokines such as TNF-α [64]. Here, we found that LPS caused the downregulation of the expression of all the vitamin K cycle genes as well as both TAM ligands Gas6 and Pros1 in microglia. By comparison, we have observed previously that LPS upregulated Axl but downregulated Mertk in microglia [34]. Therefore, the activation of inflammatory signalling in brain glia appears to suppress the local vitamin K cycle as well as negatively regulate the TAM system overall. This would therefore be expected to reduce the γ-carboxylation of tissue-derived VKDPs such as Gas6, resulting in lower amounts of functionally active Gas6 to perform its normal anti-inflammatory role. Moreover, the inflammatory suppression of Gas6/TAM signalling appears to be further compounded by the concomitant downregulation of the enzymes that carboxylate Gas6.

We also show here for the first time the functionality of the vitamin K cycle within rodent CNS cells and tissues. In both pure and mixed glial cells, we observed opposing effects of exogenous vitamin K1 and warfarin on the γ-carboxylation state of endogenous Gas6 released into the culture medium. We used vitamin K1 as an exogenous agent rather than MK-4 so as be certain of the facilitation of GGCX activity in a variety of cell types and also with the assumption that vitamin K1 is converted to MK-4 within tissues [27], thus making it an effective agent in this regard. Exogenous vitamin K1 alone did not cause an increase in carboxylated Gas6 above baseline levels, thus indicating that there was already sufficient vitamin K within the cells to enable intracellular GGCX activity. However, as an antagonist to warfarin, exogenous vitamin K1 completely nullified warfarin’s inhibitory effect on Gas6 carboxylation. These are the first observations of a functional and regulatable vitamin K cycle in brain cells.

Furthermore, we investigated the ex vivo expression and γ-carboxylation of Gas6 within brain tissues of rats that had undergone a prolonged regime of warfarin in their drinking water [44]. An analysis of these tissues revealed that the warfarin regime led to a significant reduction in Gas6 γ-carboxylation in the animal brains in situ. This finding therefore demonstrates that ingested and metabolised warfarin affects not only VKDPs in liver but also that it is able to affect VKDPs in the brain. The observed slight effect of warfarin could be due to its pharmacokinetic and pharmacodynamic profile as it traverses from oral ingestion to cross the blood–brain barrier, leaving a sufficient concentration to act significantly on brain tissue. The fact that the warfarin effect further demonstrated the functionality of the endogenous brain vitamin K cycle in vivo significantly complements previous data from in vitro studies. Furthermore, moving beyond the use of warfarin as a research tool, it is also important to consider the wider consequences of warfarin administration as an anticoagulant for certain classes of patients. For example, through also blocking the functions of extrahepatic VKDPs, warfarin might cause impairments to the neuroprotective, anti-inflammatory and pro-myelinating functions of Gas6 in the CNS. Therefore, these non-traditional effects of warfarin on VKDPs warrant further study.

## 5. Conclusions

In this study, we have shown that mouse brain and glial cells express TAM receptors and TAM ligands, as well as key components for the vitamin K-dependent γ-carboxylation of Gas6. We have observed the brain (vs. liver) expression profiles of these genes throughout early development. Inflammatory conditions resulted in a general downregulation of these genes in microglia, which coincides with the observed dysregulation of TAM signalling in neuroinflammatory diseases. Furthermore, the rodent brain has an active vitamin K cycle. Therefore, both vitamin K and warfarin have effects on Gas6 functionality within the brain, which has implications for Gas6’s role in negatively regulating neuroinflammation and stimulating repair in various neurodegenerative disorders.

## Data Availability

The datasets used and analysed during the current study are available from the corresponding author on reasonable request.

## Supplementary Materials

The following supporting information can be downloaded at: https://www.mdpi.com/article/10.3390/cells13100873/s1, Figure S1: Western blot analysis of protein expression of TAM ligands and vitamin K cycle enzymes in whole tissues and various microsome preparations from adult mouse and guinea pig, Figure S2: mRNA expression of vitamin K cycle enzyme genes (*Ggcx*, *Nqo1*, *Vkorc1*) and TAM ligands *Gas6* and *Pros1* in pure primary astrocyte cultures, following 4 h incubation with various test agents, Figure S3: Validation of Gla-Gas6 ELISA through analysis of competing effects of warfarin and vitamin K1 on cell-derived Gas6 carboxylation, Figure S4: Comparative baseline mRNA expression of the genes of interest in this study, using bulk RNA-sequencing data retrieved from the EMBL-EBI Expression Atlas search engine (https://www.ebi.ac.uk/gxa/experiments, accessed on 18 March 2024).
