# Supplementary material for: Modification of Gas6 Protein in the Brain by a Functional Endogenous Tissue Vitamin K Cycle
Source: Cells. 2024 May 18;13(10):873. doi: 10.3390/cells13100873 (PMC11119062; doi:10.3390/cells13100873)
Supplement: Supplementary file 1 [file cells-13-00873-s001.zip › cells-2948323-supplementary.pdf]

Aydin *et al*. Supplementary Figs

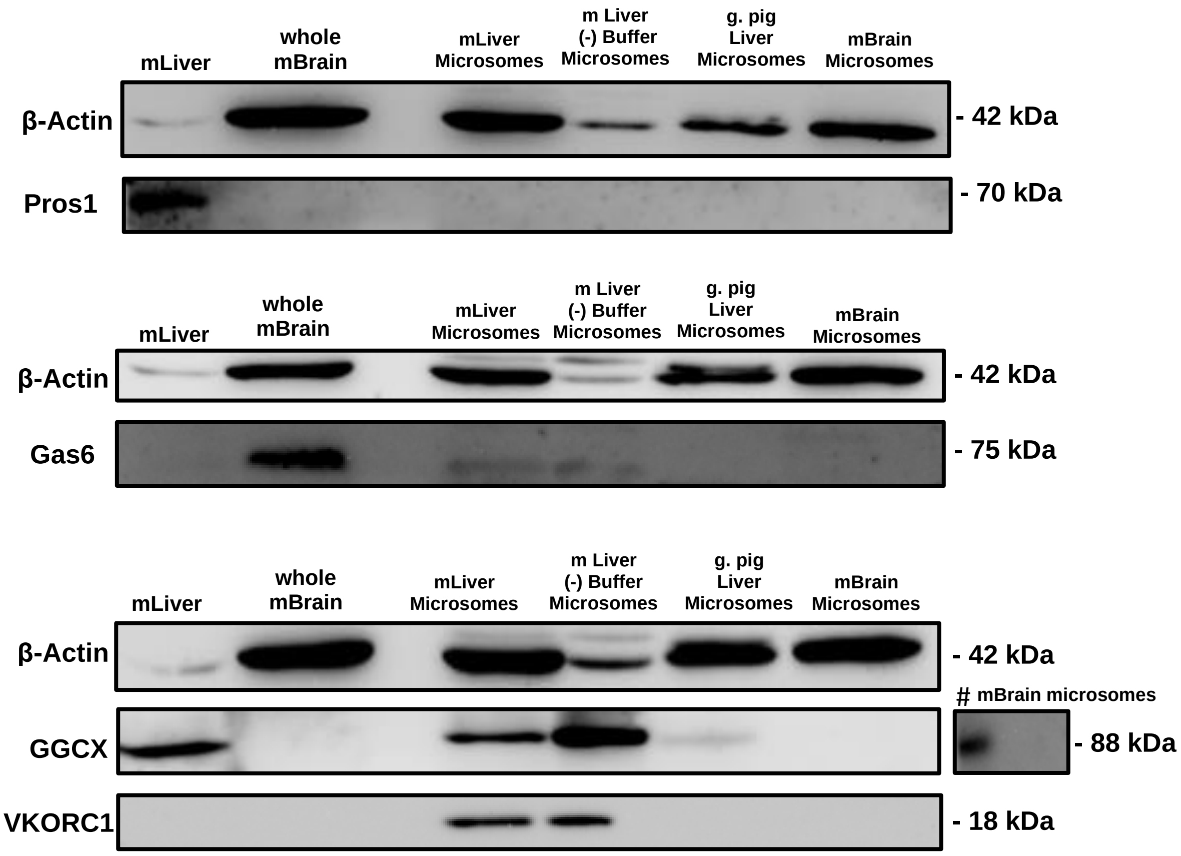

Figure S1. Western blot analysis of protein expression of TAM ligands and vitamin K cycle enzymes in whole tissues and various microsome preparations from adult mouse and guinea pig. Samples included protein extracts from adult mouse liver (mLiver), brain (mBrain), liver microsomes, and liver microsomes pellet that was not dissolved in RIPA lysis buffer (mLiver (-) Buffer Microsomes); guinea pig (g. pig) liver microsomes and adult mouse brain microsomes (mBrain microsomes). Both mouse brain and liver microsome preparations were from the same animals. #GGCX blot: after imaging the whole membrane, this section of the membrane was re-imaged separately at a longer exposure in order to detect the weaker GGCX signal in mouse brain microsomes. VKORC1 blot: this only reveals the protein at a certain, relatively low, exposure, as concentrated within liver microsome preparations, whereas its presence is masked in all other samples. Also, the antibodies used appeared not to recognise the respective proteins in guinea pig microsomes. β-Actin was used as loading control protein.

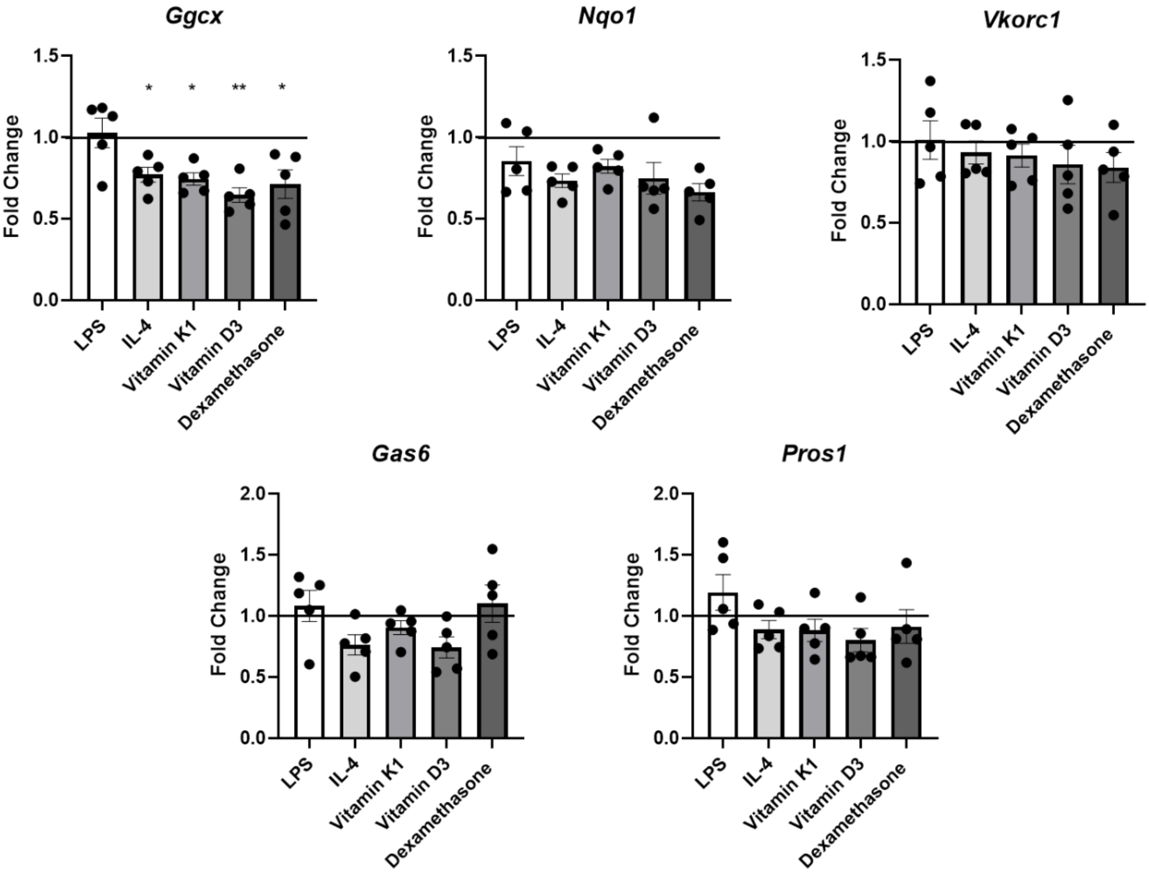

Figure S2. mRNA expression of vitamin K cycle enzyme genes (*Ggcx, Nqo1, Vkorc1*) and TAM ligands *Gas6* and *Pros1* in pure primary astrocyte cultures, following 4h incubation with various test agents. RT-qPCR data were analysed by $2^{-\Delta\Delta Ct}$ method, using *Gapdh* as housekeeping gene (mean ± SEM, n = 5 cultures (n = 4 for *Pros1*)). Statistical significance was determined using Welch's t-test with *p < 0.05, **p < 0.01 vs LPS.

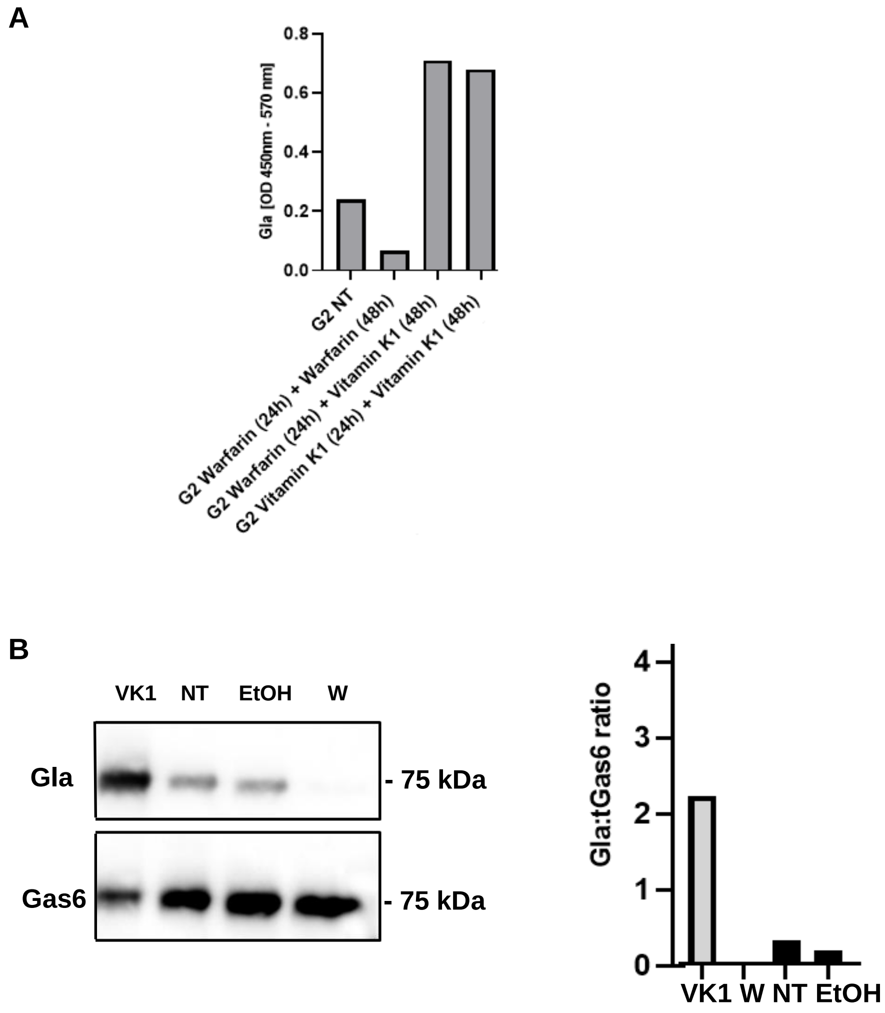

Figure S3. Validation of Gla-Gas6 ELISA through analysis of competing effects of warfarin and vitamin K1 on cell-derived Gas6 carboxylation. A. Over a total 72h period, Gas6 secreting HEK293 cells (subculture ‘G2’) were incubated in medium+vehicle only (NT) or in the presence of warfarin alone, or vitamin K1 alone, or warfarin replaced by vitamin K1 after 24h. After incubation, media were analysed by Gla-Gas6 ELISA. B. Western blot analysis of Gas6 protein in both total and γ-carboxylated forms in medium of stable Gas6 secreting HEK293 cells. Cells were not treated (NT), or treated with vehicle (ethanol; EtOH), vitamin K (VK1) or warfarin (W) for 24h. Media were analysed by successive Western blots for Gla and Gas6. Left: upper blot shows anti-Gla probed bands, which were then stripped and re-probed with anti-Gas6 (lower blot). Right: W blot densitometry analysis showing the ratio of Gla:total Gas6 band intensities. Representative experiment of 3 repeats analyses is shown.

A – RNA-Seq - Different mouse brain cell types

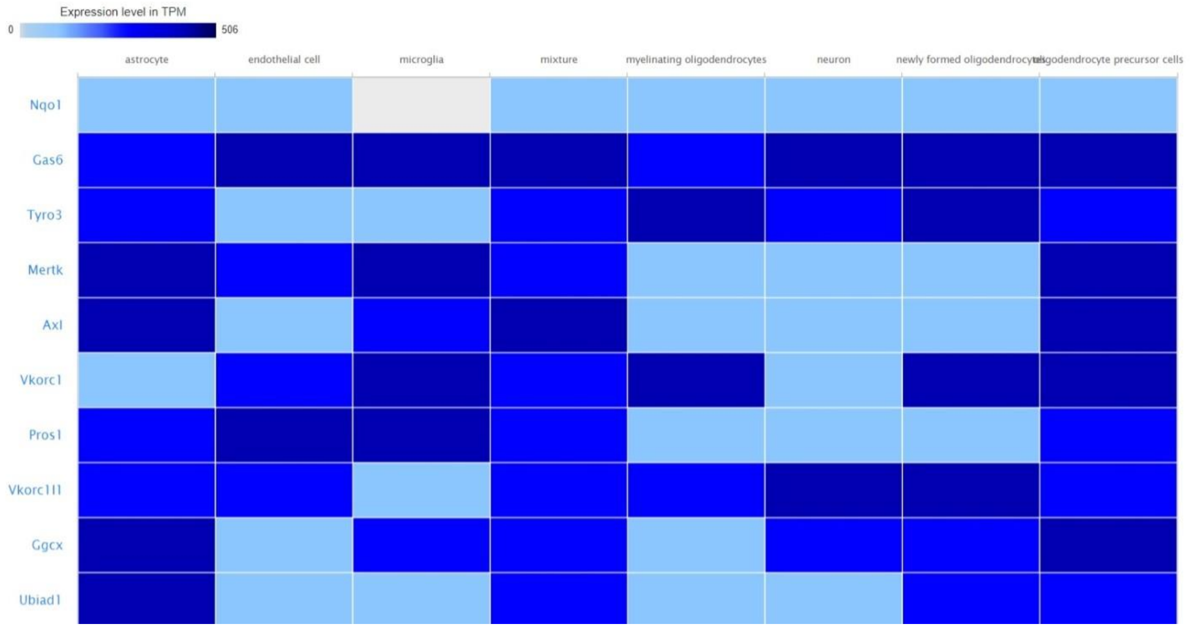

B – RNA-Seq - Mouse brain and liver during embryonic and postnatal development

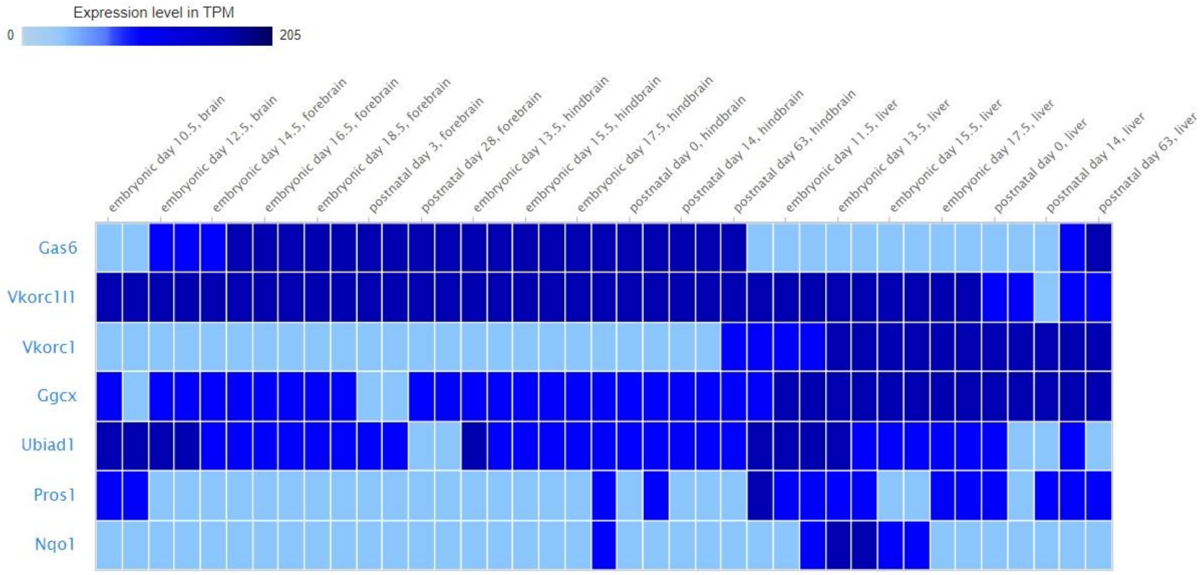

Figure S4. Comparative baseline mRNA expression of the genes of interest in this study, using bulk RNA-sequencing data retrieved from the EMBL-EBI Expression Atlas search engine (https://www.ebi.ac.uk/gxa/experiments). Genes were searched for in groups and results were filtered according to comparisons across (A) different mouse brain cell types or (B) brain, forebrain, hindbrain and liver during various developmental ages. Expression counts were extracted as transcripts per million RNA molecules (TPM) to normalize for gene length and sequencing depth across the samples.
